# Predicting the Timing and Magnitude of Tropical Mosquito Population Peaks for Maximizing Control Efficiency

**DOI:** 10.1371/journal.pntd.0000385

**Published:** 2009-02-24

**Authors:** Guo-Jing Yang, Barry W. Brook, Corey J. A. Bradshaw

**Affiliations:** 1 Jiangsu Institute of Parasitic Diseases, Meiyuan, Wuxi, Jiangsu, People's Republic of China; 2 Research Institute for Climate Change and Sustainability, School of Earth and Environmental Sciences, University of Adelaide, South Australia, Australia; 3 School for Environmental Research, Institute of Advanced Studies, Charles Darwin University, Darwin, Northern Territory, Australia; 4 South Australian Research & Development Institute, Henley Beach, South Australia, Australia; National Institute of Parasitic Diseases China CDC, China

## Abstract

The transmission of mosquito-borne diseases is strongly linked to the abundance of the host vector. Identifying the environmental and biological precursors which herald the onset of peaks in mosquito abundance would give health and land-use managers the capacity to predict the timing and distribution of the most efficient and cost-effective mosquito control. We analysed a 15-year time series of monthly abundance of *Aedes vigilax*, a tropical mosquito species from northern Australia, to determine periodicity and drivers of population peaks (high-density outbreaks). Two sets of density-dependent models were used to examine the correlation between mosquito abundance peaks and the environmental drivers of peaks or troughs (low-density periods). The seasonal peaks of reproduction (*r*) and abundance (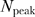) occur at the beginning of September and early November, respectively. The combination of low mosquito abundance and a low frequency of a high tide exceeding 7 m in the previous low-abundance (trough) period were the most parsimonious predictors of a peak's magnitude, with this model explaining over 50% of the deviance in 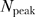. Model weights, estimated using AIC*_c_*, were also relatively high for those including monthly maximum tide height, monthly accumulated tide height or total rainfall per month in the trough, with high values in the trough correlating negatively with the onset of a high-abundance peak. These findings illustrate that basic environmental monitoring data can be coupled with relatively simple density feedback models to predict the timing and magnitude of mosquito abundance peaks. Decision-makers can use these methods to determine optimal levels of control (i.e., least-cost measures yielding the largest decline in mosquito abundance) and so reduce the risk of disease outbreaks in human populations.

## Author Summary

Mosquitoes carry several diseases that are potentially fatal to people. The risk of disease transmission is high when mosquitoes are abundant in an area, and it is therefore the job of health professionals to control or prevent mosquito outbreaks in certain areas, especially those close to human habitation. Biologists that study mosquito populations have the ability to predict peaks in mosquito population abundance by relating measures of these with environmental variables, such as tidal events and rainfall. Here we analysed data of mosquito (*Aedes vigilax*) populations from northern Australia over 15 years. We compared the highs and lows in mosquito numbers to possible drivers of these, such as tides. We found that low tide events prior to the mosquito peaks were followed by a boom in mosquito numbers. We also found the highest population growth rate is in September, which is two months earlier than the peak of mosquito abundance. Thus, following low tide events in the dry season, targeted control (such as spraying in earlier September) of mosquito breeding areas may allow for more effective control of mosquitoes close to human settlement, and therefore reduce the likelihood of disease outbreaks.

## Introduction

Only a few of the approximately 3000 mosquito species known worldwide feed on human blood [1]. Unfortunately, these human pest species are responsible for the deaths of millions of people each year by transmitting some of the deadliest-known diseases, such as malaria, yellow fever, dengue, and Rift Valley fever [2]. Different mosquito species transmit different diseases; for example, *Aedes aegypti* is the primary vector of the arboviruses dengue and yellow fevers [3],[4]; several *Anopheles* species carry different forms of the malaria protozoan parasite [5], and *Aedes vigilax* transmits Ross River and Barmah Forest virus [6],[7].

The transmission of mosquito-borne diseases is strongly linked to the abundance of the host vector [6],[8], making rigorous surveillance and control programs an essential component of disease suppression. To develop effective control measures, most studies have focused on describing and quantifying habitat associations and the particular spatio-temporal distributions of vector species. The fluctuations in mosquito abundance over time are driven both by endogenous (negative density feedback) and exogenous (stochastic environmental variation) components [9],[10]. Indeed, a recent analysis of *Ae. vigilax* population dynamics in northern Australia demonstrated that negative density feedback alone accounts for over 31% of the deviance in population growth rate, with another 40% of the deviance explained by the addition of high tide frequency, rainfall and relative humidity [10]. This combination of negative density feedback and environmental influences contribute to the characteristic oscillatory pattern of peaks and troughs in mosquito abundance over the course of a single year.

These characteristic fluctuations in abundance produce large amplitude changes in the peak timing of biting adult mosquitoes, with some extreme events occurring at wave lengths of several years. High-magnitude peaks in abundance are often correlated with the outbreak of diseases [11]–[13]. As such, the ability to predict the timing and magnitude of population peaks is an essential precursor to effective control and risk management (e.g., public warnings). A previous analysis of density fluctuations in mosquitoes in northern Australia focused on the interplay between density feedback and environmental stochasticity [9],[10]. However, there is now a need to determine what conditions lead specifically to a higher probability of high-abundance peaks that precipitate disease outbreaks. Identifying the timing and intensity of future abundance peaks is also important for maximising control efficiency because the sub-optimal and liberal application of chemical and biological pesticides has generated several ancillary problems, including excessive financial costs, the evolution of insecticide resistance via repeated exposure, safety risks for humans and domestic animals, and environmental contamination [14]–[16]. The ability to define the most effective timing and spatial configuration for applying control measures, using the smallest quantity of insecticide, will not only reduce the frequency and severity of disease outbreaks, it will alleviate many of the environmental and logistical problems associated with control and reduce costs.

There are a number of approaches developed to explain the basic causes underlying fluctuations in natural and controlled populations [17]–[20]. Various approaches have stressed the importance of weather variability, the dynamics of natural predators, prey or competitors, overcrowding, and so on [21],[22]. Yet few have been constructed to forecast simultaneously the magnitude and timing of peak population sizes in species experiencing regular density irruptions. One notable exception is the use of marginal logistic regression models to predict the timing of southern pine beetle (*Dendroctonus frontalis*) outbreaks [17]. This approach examined the influence of explanatory variables on host availability, physiography, climate, extreme weather events, and management protocols after accounting for spatial and temporal autocorrelation [17].

Modelling chaotic behaviour in population dynamics provides another approach to identify optimal intervention times. Hilker and Westerhoff [18] presented a simple method to guide management efforts in preventing crashes, peaks, or any other undesirable state in chaotic population dynamics. The method is illustrated by two examples based on captive populations of the flour beetle (*Tribolium* sp.): (1) alleviation of extinction risk in the Ricker model: 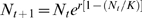 (where 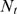 = relative abundance at time *t*, *r* [population rate of change] = log_e_(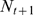/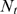) and *K* = carrying capacity), and (2) control of outbreaks in a stage-structured demographic model. First, an abundance time series of the population is analysed to identify paths that lead to crashes or outbreaks. The second step is to manipulate population abundance to force it out of the danger zone preceding either a crash (prevention of extinction) or a peak (eliminating outbreaks).

Ecological data are normally the emergent or phenomenological expression of a set of complex processes that are difficult to model mechanistically, so they cannot normally be well-represented by any one model. Model selection may help to identify which simplification of reality provides the most parsimonious explanation of the phenomenon, and multi-model inference (MMI) will draw conclusions from a weighted average over predictions made by the suite of candidate models considered [23],[24]. Previously, we used MMI for a series of phenomenological models to examine the principal drivers of population change in two well-monitored mosquito species from northern Australia [9],[10].

Using weekly relative abundance data (from CO_2_ traps), collected over 15 years in Darwin, northern Australia, we previously determined the subtle and complex interactions between density and environmental conditions [9],[10]. But this study did not examine the conditions leading to peaks in abundance over time. Here we extend our earlier approach to investigate explicitly whether the timing and magnitude of abundance peaks in *Ae. vigilax* can be determined (and so predicted) from a suite of intrinsic and measureable extrinsic components. High tide frequency and low rainfall lead to higher population growth rates in this species [10], with low rainfall in the late dry season and early wet season, in particular, facilitating mosquito breeding and subsequent abundance peaks, especially if it occurs during favourable tides. *Ae. vigilax* breeds primarily in saline to brackish wetlands along the coast, where females lay their eggs on moist mud and at the base of plants in high marshlands dominated by brackish water reeds (*Schoenoplectus* spp.) or mangroves (*Avicennia* spp. or *Bruguiera* spp.) [10].

With the highest tides able to flood the salt marshes, *Ae. vigilax* eggs are hatched immediately; however, as the tide retreats, more eggs are laid on moist substrata where they mature quickly and become drought-resistant and persist until the onset of the next high tide cycle or rain event [10]. The interval between hatchings may take weeks to months depending on the tidal patterns and rainfall, but low rainfall following the first high tide is likely to maintain sufficient soil moisture and salinity to support continued hatching [10], thus maintaining the surge of recruitment into the adult population. For the Darwin region, the first high tide>7.4 m in the late dry season (September), followed by light rains, will normally precipitate a rapid rise in the mosquito's rate population of change (*r*) and a subsequent peak in abundance in November [10],[25].

Using weekly mosquito-capture data collected over 15 years in the greater Darwin area, tropical north Australia, our specific objectives were to (i) evaluate the oscillation pattern of both the rate of population increase (*r*) and abundance over the 15 years, and (ii) estimate the relationship between peak occurrence and magnitude and changes to previously identified environmental correlates of abundance (i.e., tidal factors, rainfall and relatively humidity). Rather than simply repeat our previous work examining the processes influencing fortnightly relative abundance [9],[10], our approach was to simplify the time series and focus only on periods of highest relative abundance, indicative of peaks (and by proxy, potential disease outbreaks). Our overarching aim is to provide decision makers charged with suppressing mosquito abundance with practical advice on the timing for optimal control strategies in this and other species of mosquitoes implicated in the spread and maintenance of human infectious diseases.

## Materials and Methods

### Study area and data

Eleven monitoring locations were selected in the geographical region between 12° 22–25′S latitude and 130° 51–56′E longitude, in the swampy regions surrounding Darwin, Northern Territory, Australia [9],[10],[26]. CO_2_-baited mosquito traps [27] were checked weekly at each location by the Medical Entomology Branch of the Northern Territory Department of Health and Community Services. We assume the light intensity of the traps was the same throughout the duration of the observations. Therefore, the variation in population density is mainly a reflection of total population abundance rather than an artefact of sampling effort (see [9] for a more detailed justification).

Previously [9],[10], we argued that the comparison of monthly summary data avoids the potential confounding effects of age structure and overlapping generations, as well as providing a reasonable representation of fluctuations in population density over time. *Ae. vigilax* also exhibits only weak spatial heterogeneity within the study region, allowing us to pool population density data among the 11 trap locations as an arithmetic mean [9]. The final dataset covered 180 months from January 1991 to December 2005 (15 years).

**Figure 1:**
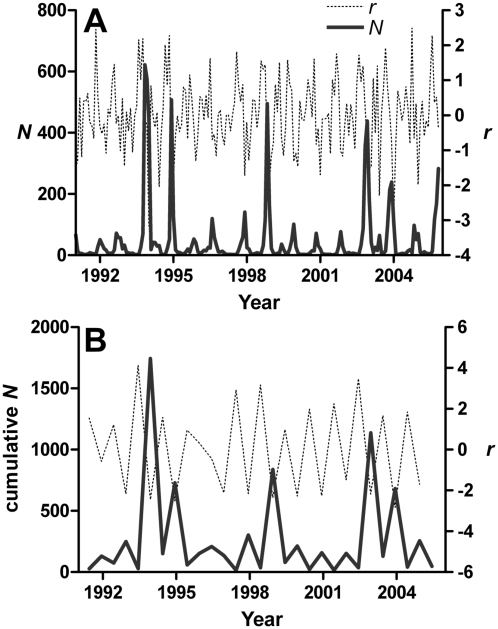
Monthly and six-month accumulated abundance ( 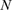
) of *Ae. vigilax* mosquitoes from 1991–2005 (principal y-axis) and the rate of population change (*r*) (secondary y-axis). A. Monthly dataset. B. Six-monthly reduced dataset in the periods of abundance peaks and troughs.

A visual examination of the oscillations of mosquito abundance time series clearly shows the semi-regular occurrence of high-abundance ‘peaks’ interspersed with low-abundance ‘troughs’ (Fig. 1A). Our *a priori* hypothesis is that environmental variation can be used to predict the frequency and magnitude of abundance peaks after taking density feedback into account. To test this hypothesis, we split the relative abundance time series for *Ae. vigilax* from 1991 to 2005 into periods representing seasonal periodicity: relatively high (September–February), and low (March–August) abundance periods (28 seasonal intervals over 15 years) (Fig. 1B). For each six-month period, the numbers of mosquitoes were summed in subsequent analyses. Monthly population growth rates (*r*) were taken as *r_t_* = log_e_(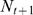/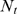), where 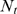 is the measure of relative abundance at time point *t*.

### Environmental conditions

**Figure 2:**
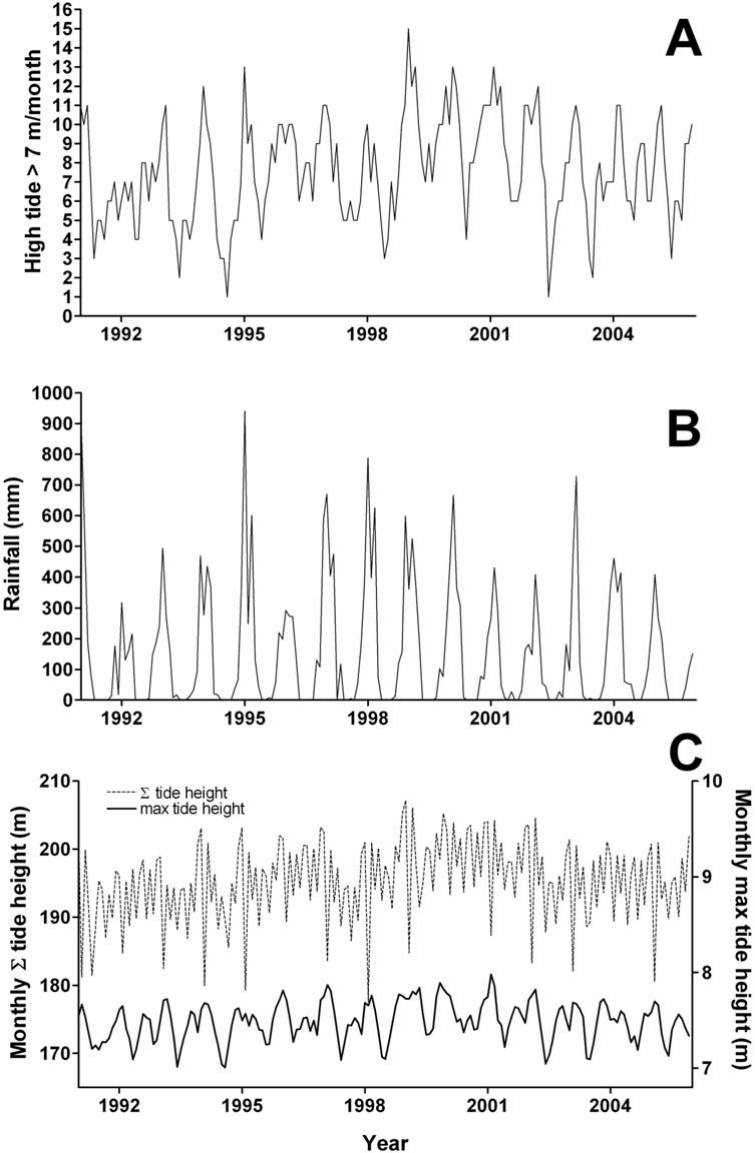
Temporal plots of environmental variables measured from 1991–2005. A. Number of high tides>7.0 m per month from 1991–2005. B. Total rainfall (mm) from 1991–2005. C. Monthly accumulated tide height (principal *y*-axis) and maximum tide height per month (secondary *y*-axis).

Monthly environmental data covering the same interval as the mosquito abundance dataset were provided by the Australian Government Bureau of Meteorology (www.bom.gov.au). Frequency of high tide (Fig. 2A), rainfall (Fig. 2B) and relative humidity have all been previously identified as correlated with the rate of population change for this species, with the strongest explanatory variable being high tide frequency [10]. For the question at hand, we also considered two additional descriptors of tidal patterns that measure the magnitude of tidal influence in these ephemeral saltwater habitats: (1) accumulated tide height per month, and (2) maximum tide height per month (Fig. 2C).

### Periodicity

Our previous work [10] determined that the ecosystem's carrying capacity is relatively invariant compared to *Ae. vigilax*'s response to fluctuating extrinsic conditions. Therefore, periodicity in relative abundance (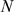) represents mainly the population's response to environmental conditions promoting egg hatching, larval development and adult dispersal. Periodicity in *r*, on the other hand, indicates variation in the population's relative distance from carrying capacity, and so is more indicative of internal feedback mechanisms. We therefore examined periodicity in both properties to determine the oscillatory dynamics of *Ae. vigilax*.

Let 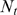 and 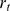 be the monthly mosquito abundance and population growth rate, respectively. We fitted a seasonality model through the harmonic curves of 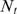 and 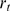 of the following form:
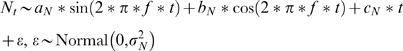
(1)

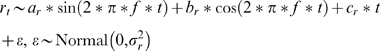
(2)which can be simplified to:
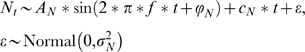
(3)

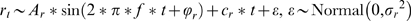
(4)Here, amplitude 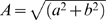 (*a* and *b* are the estimated coefficients for the sine and cosine terms in Equations 1 and 2); 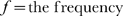 (1 month/12 months); 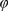 is the phase 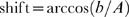, and 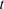 defines any linear temporal trends in the response. The month where the seasonal peak occurs is then:
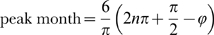
(5)where *n* = is the number of the cycle. The fundamental time-series modelling tool for spectral analysis is the periodogram, which is based on the squared correlation between the time series and sine-cosine waves of frequency. Periodograms were analyzed using the spec.pgram function in the *R* Package v2.4.0 [28].

### Predicting abundance peaks

Two sets of models were used to examine the correlation between mosquito abundance peaks (Fig. 1B) and the environmental drivers considered:
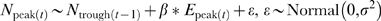
(6)

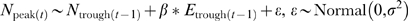
(7)where 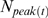 and 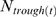 denote total mosquito population size during the identified peak and trough intervals, respectively, 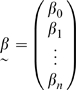 is the vector of coefficients for the *n* environmental drivers considered, and 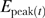 and 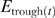 represent the vector of environmental drivers (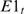, 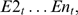) during the peak and trough intervals, respectively. Our previous identification of a strong density feedback component in the oscillation dynamics of this mosquito species [9] argued for the inclusion of the previous 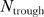 value as an explanatory covariate (Equations 6 and 7). The mid-point of the trough interval (6-month duration) occurs exactly 6 months prior to the identified peak date given the definition of the ‘trough’ and ‘peak’ periods and the identification of dominant 12- and 6-month periodicities (see Results).

We contrasted a total of 44 models comprising various combinations of the terms of interest, fitted using maximum-likelihood estimation. All analyses were done using the *R* Package [28]. Model comparisons were based on multi-model inference (MMI) using Akaike's Information Criterion corrected for small sample size bias (AIC*_c_*) as an estimate of Kullback-Leibler (K-L) information loss [24],[29]. The difference between the model's criterion and the top-ranked model (ΔAIC*_c_*) and the relative model weights (*w*AIC*_c_*) were calculated. Thus, the strength of evidence (*w*AIC*_c_*) for any particular model varies from 0 (no support) to 1 (complete support) relative to the entire model set. For each model we also calculated the % deviance explained (%DE) as a measure of the model's goodness-of-fit, the predicted *R* ^2^ as a measure of the % variance explained, and the leave-one-out cross-validation prediction error (C-Vε) based on the cv-glm command in the *R* Package [28] to validate the robustness of the predictions.

## Results

### Periodicity

**Figure 3:**
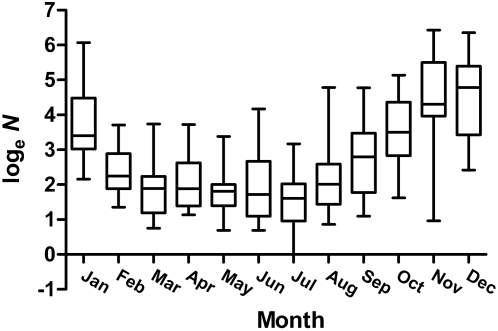
Box-and-whisker plots showing the monthly variation in population density of *Ae. vigilax* from 1991–2005. Monthly values show the median, 10 and 90 percentiles, and the minimum and maximum observations.

Figure 1A shows the time series of monthly mosquito abundance (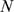) and population rate of change (*r*) between 1991–2005, clearly depicting the strong seasonality in the temporal pattern of both 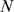 and *r*. In Figure 1B, the reduced peak-trough dataset is presented. For the 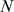 time series, amplitude (*A*) of the time-series curve was 62.98 trap-caught individuals: fitted mosquito abundance fluctuated between 0 and 125.96 per trap (2*A*). The seasonal peak of the mosquito population occurs in early November (month 11.3); the September–February period is considered the high-abundance season, and March–August the low-abundance season (Fig. 1B and 3). The coefficients for the sine and cosine elements in the 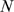 periodicity model were −15.95 and 60.93, respectively. We found weak evidence for a slight uptrend in 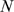 (coefficient = 0.06242 [SE = 0.116]) over time between 1991–2005.

**Figure 4:**
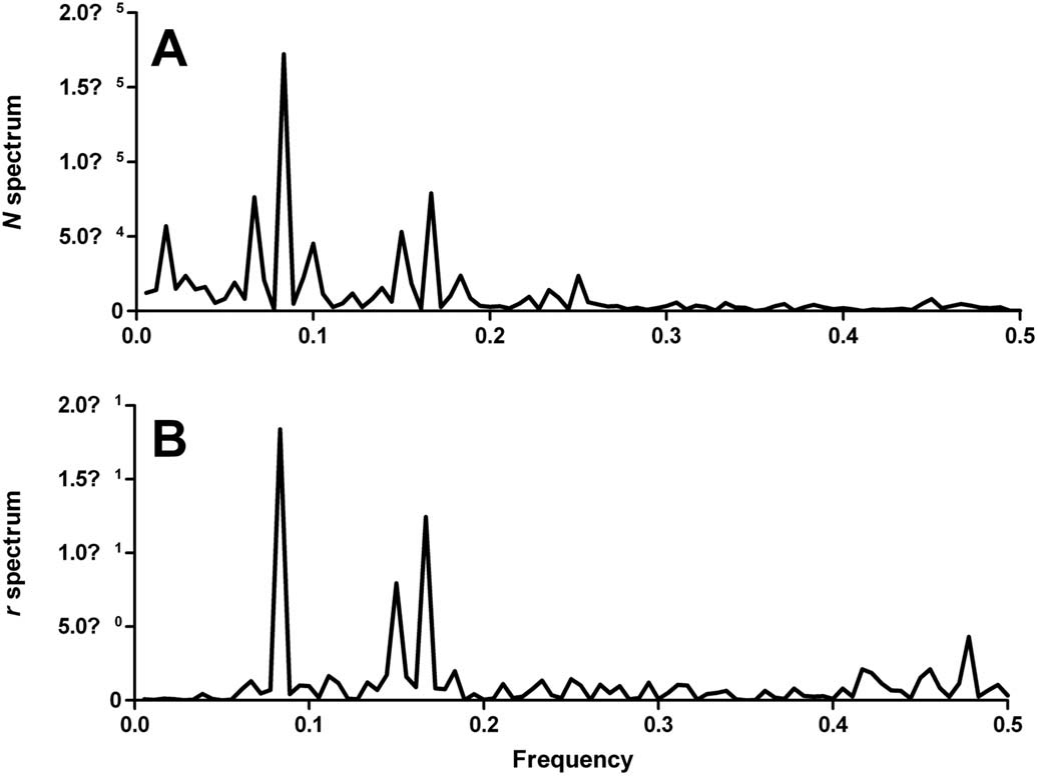
Estimated periodogram of the de-trended time-series of monthly abundance and population change (*r*) of *Ae. vigilax* in Darwin, 1991–2005. A. monthly abundance of *Ae. vigilax*. B. monthly population change rate (*r*) of *Ae. vigilax*.

For *r*, the amplitude (*A*) of the time series = 0.66, with the seasonal peak occurring at the beginning of September (9.0), which is over two months earlier than the expected mosquito abundance peak. The periodograms of 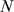 and *r* (Fig. 4) confirm the seasonality with strongest periodicities of 12 months (largest peak with frequency 1/12 = 0.08), followed by a weaker periodicity of 6 months (second highest peak with frequency 1/6 = 0.17). The coefficients for the sine and cosine elements in the *r* periodicity model were −0.58 and −0.32, respectively.

### Predicting abundance peaks

**Figure 5:**
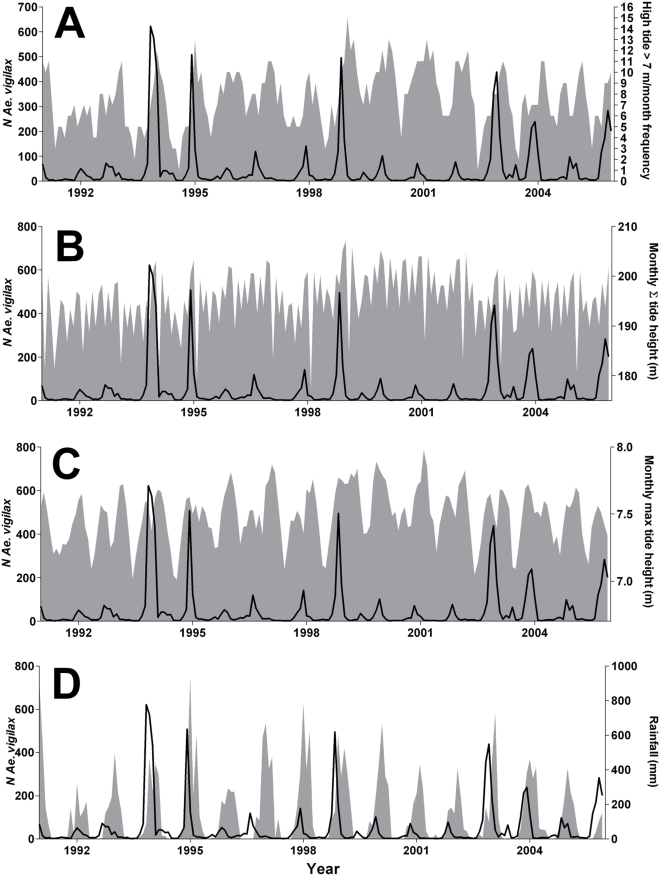
Monthly abundance of *Ae. vigilax* (black lines; principal *y*-axis) in relation to environmental correlates (secondary *y*-axis). A. Frequency of high tides>7.0 m per month (grey); B. Accumulated tide height per month (grey); C. Maximum tide height per month (grey); D. Total rainfall (mm) per month (grey).

Among the two sets of models, the frequency of high tides above 7 m per month in the previous trough interval had the strongest *w*AIC*_c_* support for explaining variance in 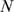 during the high-abundance period (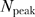) after accounting for the previous trough's abundance (Table 1). In the top-ranked model,
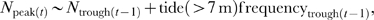
the first (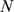
_trough(*t*−1)_) and second (tide>7 m frequency) term's coefficient estimates were −0.98 and −39.07, respectively. This demonstrates that the combination of low mosquito abundance and a low frequency of high tide exceeding 7 m in the previous low-abundance (trough) period were the most parsimonious predictors of a peak's magnitude (Fig. 5A), with the model explaining over 50% of the deviance in 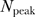. This was also confirmed by the leave-one-out cross-validation. The top *w*AIC*_c_*-ranked model also had the lowest prediction error (Table 1). Model weights were also relatively high for those including maximum tide height, accumulated tide height or total rainfall in the trough period (Table 1, Fig. 5B–D), with high values in the trough correlating negatively with the onset of a high-abundance peak. For the second highest-ranked model including 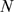
_trough(*t*−1)_, tide frequency and maximum tide height (Table 1), the coefficients were −0.69, −76.92 and 4281, respectively. For the third highest-ranked model including 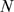
_trough(*t*−1)_, tide frequency and accumulated tide height (Table 1), the coefficients were −1.41, −68.75 and 20.38, respectively.

**Table 1:** The 13 highest-ranking correlative models linking abundance of *Ae. vigilax* during the peak (outbreak – high density) periods to plausible environmental drivers, with 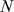
_trough_ (low density) being a control variable to account for density-dependent recovery due to compensatory population regulation [9].

Model	*LL*	*k*	AIC*_c_*	ΔAIC*_c_*	*w*AIC*_c_*	C-Vε	%DE	*R* ^2^
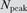 ∼ 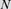 _trough_ *+T.7*	−100.982	4	214.408	0.00	0.316	171749.4	50.1	0.41
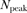 ∼ 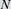 _trough_ *+T.7+T.M.*	−98.758	5	215.016	0.61	0.233	172387.8	63.7	0.53
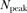 ∼ 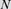 _trough_ *+T.7+T.S.*	−99.065	5	215.630	1.22	0.171	173777.4	62.0	0.51
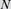 ∼1	−105.843	2	216.778	2.37	0.097	249122.7	0.0	0.00
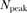 ∼ 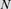 _trough_ *+T.7+Rain*	−100.411	5	218.322	3.91	0.045	181632.6	54.0	0.40
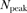 ∼ 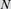 _trough_ *+T.7+RH*	−100.678	5	218.855	4.45	0.034	206598.1	52.2	0.38
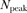 ∼ 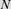 _trough_	−105.763	3	219.925	5.52	0.020	278135.2	1.2	−0.07
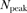 ∼ 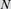 _trough_ *+Rain*	−104.037	4	220.518	6.11	0.015	274945.2	22.8	0.09
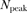 ∼ 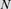 _trough_ *+T.7+T.S.+RH*	−98.420	6	220.840	6.43	0.013	222182.8	65.4	0.50
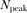 ∼ 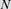 _trough_ *+T.M.*	−104.251	4	220.947	6.54	0.012	254994.1	20.4	0.06
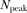 ∼ 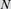 _trough_ *+T.7+T.M.+RH*	−98.698	6	221.395	6.99	0.010	229772.0	64.0	0.48
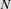 ∼ 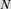 _trough_ *+T.S.*	−104.544	4	221.533	7.12	0.009	279307.5	16.9	0.02
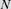 ∼ 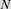 _trough_ *+Rain (p)*	−104.656	4	221.757	7.35	0.008	287998.4	15.6	0.003

The models incorporating environmental conditions in the peak period are indicated by ‘(p)’. The environmental variables include: frequency of high tide>7 m per season (T.7), accumulated tide height per season (T.S.), average of monthly maximum tide height per season (T.M.), total rainfall (Rain) per season and relative humidity (RH). Shown are *LL* = maximum log-likelihood, *k* = number of parameters, AIC*_c_* = Akaike's Information Criterion corrected for small sample sizes, ΔAIC*_c_* = the difference between the model AIC*_c_* and the minimum AIC*_c_* in the set of models, and AIC*_c_* weights (*w*AIC*_c_*) = the relative likelihood of model *i*, C-Vε = leave-on-out cross-validation prediction error, %DE = % deviance explained by the model and *R* ^2^ = predicted *R* ^2^ (this can take negative values when the fit is ranked lower than the intercept model).

**Figure 6:**
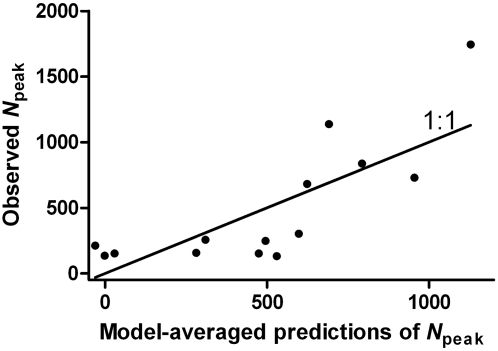
Model-averaged predictions based on the three most highly ranked models to predict mosquito abundance during the peak period (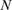
_peak_) (see Table 1) relative to observed abundances. Also shown is the optimum 1∶1 relationship between observed and predicted peak abundances.

We used the three most highly ranked models (Table 1), accounting for 72% of the model weights (the fourth-ranked model was the intercept-only model (no predictors), so this was excluded along with the remaining models that accounted for ∼18% of the remaining weights), to calculate a model-averaged 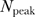 prediction (Fig. 6). Based on these results, it can be seen that predictions mimic observed values rather well. With the primary applied aim of predicting the highest-abundance peaks, our method thus provides an adequate tool for managers to prepare and mitigate mosquito outbreaks.

## Discussion

*Ae. vigilax* is a particularly notorious and heavily controlled mosquito species in Australia because of its role in the spread and maintenance of several serious infectious human diseases such as Ross River and Barmah Forest fevers [30],[31]. Indeed, these two diseases were the most common mosquito-borne diseases reported in Australia in 2005–2006 [32], with over 5000 cases of Ross River fever reported annually. Previous studies suggested that human infection rates are related to the appearance of large numbers of adult *Ae. vigilax* [8], with the recommendation that targeted control of adults in disease-prone areas could reduce the number of cases substantially. As such, identifying the environmental and biological precursors that herald the onset of peaks in mosquito abundance provide health and land-use managers the capacity to predict the timing and distribution of the most efficient and cost-effective mosquito control. Our results presented here on the amplitude and timing of outbreaks, when combined with the more general previous work on intrinsic and environmental determinants of mosquito population dynamics [9],[10], clearly demonstrate that a relatively simple set of conditions – low abundance of adult mosquitoes in the trough season, coupled with a low frequency of high tide and low rainfall – can predict peaks in mosquito abundance and potentially outbreaks of human disease with sufficient reliability to be a useful decision-making tool for managers.

The rate of population change (*r*) has been widely used to model the way in which animal or plant populations change with time [33],[34]. We found that the low relative abundance during the dry season months were generally indicative of impending population peaks. Hence, the development of ideal environmental conditions (see below), coupled with rapidly rising abundances, indicate the optimal times to apply control in swamps surrounding human settlements to minimize the magnitude of subsequent peaks. Further, we found that maximum *r* generally occurred around two months prior to the appearance of abundance peaks, so that regular monitoring should provide managers with ample preparatory time in which to organise and implement widespread control (e.g., in September for *Ae. vigilax* in Darwin).

Our results suggest that control operations for *Ae. vigilax* in northern Australia should target the period approximately two months leading up to an eventual peak, when ‘trough’ abundances are relatively low and few recent high tide events have occurred. Thus, following low tide events in the dry season, targeted control such as spraying of mosquito breeding swamps in early September will allow for more effective control close to human settlements. Another element to optimise reduction efficiency is the spatial configuration of control measures. In Darwin, the bacterial larvicide, *Bacillus thuringiensis* var. *israelensis* (B.t.i), and temephos (an organophosphorus insecticide), are widely used for larval control and are broadcast via ground and aerial (helicopter) operations [35]. Adulticides are generally less effective due to the ability of adult mosquitoes to disperse over wide areas, including human settlements (e.g., [36]). Control is often done at a local, administrative scale, with the choice of spatial configuration depending traditionally on accumulated trial-and-error knowledge rather than any systematic analysis of spatial data. Many other mosquito control studies have placed emphasis on determining the optimal spatio-temporal distribution of adult mosquitoes [8],[37],[38]. We suggest that such approaches should also be applied to *Ae. vigilax* larvae to improve control efficiency further.

Perhaps counter-intuitively, we found that the magnitude of mosquito peaks was negatively associated with the frequency of high tide above 7 m and maximum high tide over the previous season of low abundance. This may be explained by considering the chaotic population dynamics typical of oscillating populations. Both aquatic (e.g., algal blooms) [39] and terrestrial (e.g., insect outbreaks) [40] studies have found that population crashes are often preceded by an immediate peak in population size, and vice versa. For instance, in a series of microcosm experiments, Hilker and Westerhoff [18] showed that the addition of adult flour beetles (*Tribolium castaneum*) immediately prior to the occurrence of an anticipated population peak reduced the probability that one would occur. This is generally attributed to the strong negative feedback on the survival and perhaps fertility of individuals invoked by intra-specific density-related competition and predation [9],[18]. In the case of *Ae. vigilax*, the lack of high tides during the low-abundance phase necessarily impedes the hatching of eggs, leading to low initial population densities as the season progresses. The low densities and accumulation of eggs therefore provide the opportunity for *en masse* hatching and high post-hatching survival once conditions become favourable, leading inevitably to high-magnitude peak in adult abundance.

In conclusion, we have shown that basic environmental monitoring data can be coupled to relatively simple density-feedback models to assist in predicting the timing and magnitude of mosquito peaks which lead to disease outbreaks in human populations. Our results demonstrate this capacity for the control of a mosquito species in northern Australia which is responsible for many cases of infectious viral diseases. We propose that our model can be applied to any other mosquito populations where the appropriate monitoring and environmental data are available, so that optimal levels of control (i.e., least-cost measures bringing the largest decline in mosquito abundance) can be implemented to alleviate suffering and save lives and money in tropical regions worldwide.
